# Examining the Characteristics and Applications of Mesenchymal, Induced Pluripotent, and Embryonic Stem Cells for Tissue Engineering Approaches across the Germ Layers

**DOI:** 10.3390/ph13110344

**Published:** 2020-10-26

**Authors:** Caitlin Priester, Amber MacDonald, Madhu Dhar, Austin Bow

**Affiliations:** 1 Department of Animal Science, University of Tennessee, Knoxville, TN 37998, USA; cprieste@vols.utk.edu; 2 Department of Large Animal Clinical Sciences, College of Veterinary Medicine, University of Tennessee, 2407 River Drive, Knoxville, TN 37996, USA; amacdon4@vols.utk.edu (A.M.); mdhar@utk.edu (M.D.)

**Keywords:** stem cells, mesenchymal stem cells, induced pluripotent stem cells, embryonic stem cells, regenerative medicine, mesoderm, ectoderm, endoderm, biomaterials, regenerative medicine

## Abstract

The field of regenerative medicine utilizes a wide array of technologies and techniques for repairing and restoring function to damaged tissues. Among these, stem cells offer one of the most potent and promising biological tools to facilitate such goals. Implementation of mesenchymal stem cells (MSCs), induced pluripotent stem cells (iPSCs), and embryonic stem cells (ESCs) offer varying advantages based on availability and efficacy in the target tissue. The focus of this review is to discuss characteristics of these three subset stem cell populations and examine their utility in tissue engineering. In particular, the development of therapeutics that utilize cell-based approaches, divided by germinal layer to further assess research targeting specific tissues of the mesoderm, ectoderm, and endoderm. The combinatorial application of MSCs, iPSCs, and ESCs with natural and synthetic scaffold technologies can enhance the reparative capacity and survival of implanted cells. Continued efforts to generate more standardized approaches for these cells may provide improved study-to-study variations on implementation, thereby increasing the clinical translatability of cell-based therapeutics. Coupling clinically translatable research with commercially oriented methods offers the potential to drastically advance medical treatments for multiple diseases and injuries, improving the quality of life for many individuals.

## 1. Introduction

Stem cells are immature cells capable of self-renewal and differentiation into functional cell types [1]. The two primary classifications for stem cells denote origin (embryonic or adult tissue derived) and differentiation potency, the latter of these indicating the potential lineages to which a given cell can mature [2,3]. The utility of stem cells can best be characterized by both this differentiation metric and their self-renewal capability, which in combination offers a uniquely powerful biological tool for the development of treatments targeting a wide array of injuries and diseases. To maximize effective application of stem cells in emerging therapies, clear definitions of the origin and potential of the various cell types available is required [4]. Three primary types of stem cells are mesenchymal stem cells (MSCs), induced pluripotent stem cells (iPSCs), and embryonic stem cells (ESCs). These cells mainly differ by differentiation potential which impacts applicability in regenerative medicine. 

MSCs, isolated from adult tissues such as bone marrow and adipose tissue, demonstrate a readily accessible cell source with versatile differentiation potential [5]. Considered multipotent, these cells are innately capable of differentiating to lineages associated with the mesoderm germ layer and therefore constitute an attractive option for treating bone, cartilage, and muscle injuries [6]. Additionally, MSCs can mature toward lineages of other germ layers when given appropriate external stimulation. Therefore, MSCs are a major focus for development of clinically translatable therapeutic applications. In contrast to the multipotency of MSCs, the pluripotency of iPSCs and ESCs permits these cells to readily differentiate to lineages of mesodermal, ectodermal, and endodermal layers [7]. This highly dynamic maturation potential represents a uniquely powerful tool for regenerative medicine as it provides a therapeutic agent capable of application in a wide array of injuries and diseases [8]. Furthermore, pluripotent cells have also seen pronounced research application in chimeric creation and organoid synthesis, both of which have transformative implications for the future of medicine [9]. However, this high degree of differentiation potency raises concerns regarding teratomas formation, particularly in the application of iPSCs as these cells must be reprogrammed from their initial somatic tissue [10,11].

Stem cells complement their applications in the field of regenerative medicine, ranging from simple injection of cells at a lesion site to seeding of cells within intricate scaffold designs for implantation. Biomaterials have a critical role for providing a platform capable of not only delivering the stem cell payload, but also maintaining an environment for proliferation after implantation [12,13,14]. For this reason, a plethora of scaffold compositions incorporating organic and/or synthetic constituents have been utilized in conjunction with stem cells to generate effective treatments for injuries to target tissues [15]. However, these inconsistencies in cell delivery method, as well as the lack of an ideal dosing metric, raise challenges for direct comparisons of study data [16,17]. The employment of tracking mechanisms, such as fluorescent cells, and powerful analytical tools, capable of assessing transcriptomic and metabolomic profiles, may improve the ability to compare independent studies, thereby permitting the formation of a more standardized approach to stem cell delivery.

The objective of this review is to concisely address the fundamental aspects of these three types of stem cells, addressing the potential advantages and limitations of each. Particular focus is given to current and emerging applications for each stem cell type in developing treatments for injuries and diseases. These applications will be limited to therapies involving implantation of intact cells and cell-seeded biomaterials and will not include those utilizing cell-produced bioactive agents such as secreted proteins, microvesicles, and other small molecules. By providing a summarized portfolio describing the characteristics and potential roles for each of these stem cell types, the review intends to stimulate future innovations and advancements in the field of regenerative medicine.

## 2. Stem Cell Basics

As previously discussed, stem cells are characterized by their propensity for self-renewal and capacity to differentiate to functional cells. The main differences among stem cell types relate primarily to the differing degrees of differentiation potency. To further examine the core aspects of these stem cell types, a wealth of technologies has been employed to examine and classify the key attributes of each, as well as to highlight their functional applications. Such tools as RNA sequencing, mass-spectrometric metabolite analysis, flow cytometry, immunofluorescence, and Western blotting techniques have been utilized to generate profiles of the transcriptome, metabolome, and proteome with high degrees of accuracy. Furthermore, methods of assessing differentiation potential, including special staining protocols and gene expression, can provide essential data associated with the “stemness” of a cell culture. From this impressive array of data and methods it has become evident that the most vital aspect for research of these undifferentiated cells is consistency. The ability to generate cell populations that display consistent and predictable patterns, both in growth and differentiation, is an essential foundation for developing effective clinically translatable therapeutics utilizing either stem cells alone or as a biological additive to a biomaterial [18]. The importance of this standardization of culture methods is further observed when considering the inherent modifications to the native state of these undifferentiated cells upon exposure to a tissue culture environment. The conditions associated with in vitro culturing are substantially different from the highly dynamic nature of an in vivo environment and results in alterations of basal expression of genes and proteins. In an effort to reduce this variation and increase clinical translatability, an emphasis on minimal manipulation of isolated cells has been implemented in regulatory considerations by the FDA. For this reason, processes such as cell sorting by specific surface markers for MSCs may be less effective than expansion of initially heterogeneous cultures following enzymatic extraction of cells from tissue. These cultures may better represent populations found in vivo as supporting cells are not removed, as well as cells not being exposed to the mechanical stresses associated with sorting. Likewise, expansion and preservation of cells at early passage can reduce the alterations in stem-like properties of populations. 

The following sections will focus on defining the established profile characteristics of each stem cell type by exploring the source tissues and processes associated with establishing cultures, the differentiational potency, and the methods associated with verifying that cultures are viable and reproducible.

### 2.1. Mesenchymal Stem Cells

**Figure 1:**
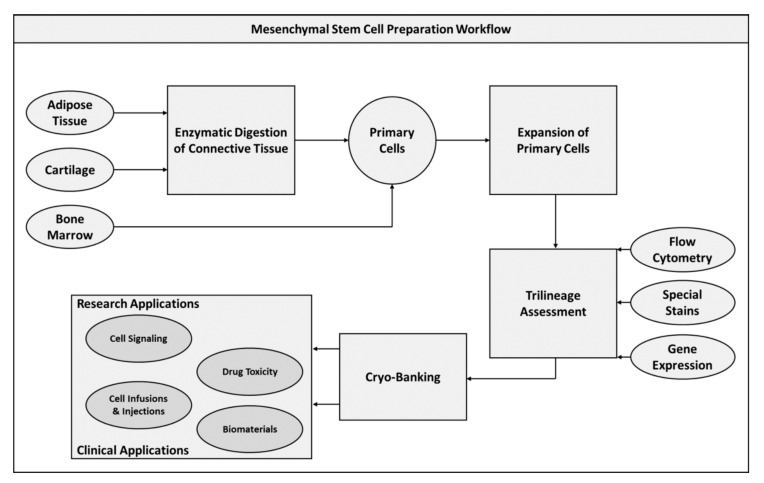
Mesenchymal stem cell preparation workflow diagram.

MSCs, as previously described, are undifferentiated cells derived from tissues of the mesodermal germ layer, with the most common sources being from bone marrow and adipose tissue. The use of nomenclature for MSCs has continued to be a point of debate with some researchers insisting that the term “mesenchymal stromal cells,” is more appropriate. As MSCs are isolated from various tissues of the mesodermal layer, they are predisposed to differentiate to tissue of the mesoderm and require specific environmental conditions for other maturations, for this reason the classification of such cells as truly “stem” can be called into question [19]. This is all the more complicated by the widespread use of the term “stem cells” by commercial entities. For the purposes of this review, MSC will indicate cell populations that have been rigorously characterized and found to express specific surface markers, as well as be capable of readily differentiating to all lineages within the mesoderm layer. These cells have been largely explored due to their versatility and ease of collection and availability, contributing to the advancement of both regenerative medicine and experimental biology [20]. Originally harvested from bone marrow in the 1960s, MSCs have been characterized as cells that express a collection of surface markers, are capable of proliferating with at least one undifferentiated daughter cell, and can differentiate to lineages within the mesodermal lineage. Regarding surface markers, MSCs should express cluster of differentiation (CD) 90, CD73, CD105, and lack of expression of CD45, CD34, CD14, CD11b, CD79α, CD19, and human leukocyte antigen-antigen D related (HLA-DR) surface molecules [21,22]. Negative markers distinguish MSCs from endothelial or hematopoietic progenitor cells [23]. Expansion of cultures from primary tissue is conducted by periodic media changes to remove nonadherent cells, with enzymatic passaging performed to prevent overcrowding of flasks. The expanded adherent cells should be capable of differentiating to osteogenic, chondrogenic, and adipogenic lineages, which can be confirmed through a tri-lineage staining assessment [20]. This stain series applies the colorimetric agents Alizarin Red, Alcian Blue, and Oil Red O to detect calcium (osteo), glycosaminoglycans (chondro), and lipid (adipo) content [24,25]. Additionally, bone marrow-derived MSCs (BM-MSCs) have shown a capacity for differentiation to both skeletal muscle and cardiac tissues [26,27,28,29]. After characterization of cultured cells, the expanded population can then be cryo-preserved for future applications (Figure 1).

In examination of the BM-MSCs and adipose-derived MSCs (AD-MSCs), it should be noted that, while there are similarities in collection, expansion, and potential of these cell types, there are key differences in processing and innate differentiation capacity. This differentiation potential difference is a result of undifferentiated cells being predisposed to mature to the tissue type in which they reside. BM-MSCs have been extensively studied for bone regeneration, but major limitations prevent clinical use as extraction requires an induced bone defect from either the hip, rib, or facial bones [30]. Not only are these high risk and painful procedures, but BM-MSC extraction often fails to obtain a sufficient number of cells for clinical use. Human BM-MSCs isolated from the iliac crest showed an estimated concentration of 0.001–0.01% after eliminating other cell types [31]. Other sources describe yields as low as only a few hundred cells per milliliter when clinical injection would require millions of cells [32,33]. Additionally, the osteogenic potential of BM-MSCs was shown to decline with age, suggesting BM-MSCs may be an impractical stem-cell candidate for age-related bone conditions [34]. These limitations have turned attention to adipose-derived MSCs (AD-MSCs) as an alternative resource for bone repair and regeneration. 

The AD-MSC population is highly concentrated with estimated yields being 100–500-fold times higher than BM-MSCs [32]. Additionally, a large amount of fat tissue (averaging about 2 L) can be extracted per liposuction procedure which would normally be discarded as waste, thereby providing a MSCs cell source that is easily accessible and requires less invasive procedures to extract [35]. Furthermore, their multipotent nature provides the capacity for differentiation to osteoblast, chondrocyte, and the myocyte maturations, with the myogenic lineages further subdivided to skeletal, cardiac, and smooth muscle cells [36,37]. These cell fates are primarily controlled by the master transcription factors MyoD (myoblast lineage), Sox9 (chondrogenic lineage), PPARγ (adipogenic lineage), and Runx2 (osteogenic lineage) [38]. The versatility of AD-MSCs can therefore be harnessed with relative ease and readily expanded to facilitate both research and clinical applications.

MSCs are a subcategory of multipotent stem cells, which are cells capable of differentiating to a specific set of tissues, as opposed to pluripotent stem cells that can mature to all adult tissue types. Other examples of multipotent stem cells are skeletal stem cells (SSCs), hematopoietic stem cells (HSCs), neuronal stem cells (NSCs), and intestinal stem cells [7,39,40]. However, some of these cell types present challenges with regards to extraction and isolation, thereby making MSCs an attractive focus, as these cells can be stimulated to mature toward other multipotent cell lines. Notably, the source tissue from which MSCs are isolated has been demonstrated to result in initial cell populations with varying basal gene expression, thereby resulting in variances in differentiation potential. Additionally, MSCs have the ability to avoid immune rejection following implantation, with studies demonstrating the potential of these undifferentiated cells to suppress T-cell proliferation and extend the survival time of cell-seeded grafts [41,42]. Furthermore, these cell populations have shown a capacity for immunoregulation through regulating surface expression of human leukocyte antigen-I (HLA-I), a transmembrane glycogprotein present in nucleated cells. The interaction of CD8-expressing T-cells with HLA-I serves as a mechanism for the recognition and elimination of foreign cells through cytotoxic functions. Therefore, the modulation of HLA-I surface expression by implanted autogenic and allogenic MSCs represents a powerful biological tool for stimulating tissue repair without eliciting transplant rejection [43]. 

### 2.2. Induced Pluripotent Stem Cells

iPSCs, originally developed as an alternative to ESCs in order to combat the ethical concerns surrounding their use, have been labeled as one of the most promising tools for the advancement of regenerative medicine. Generated from reprogramming of somatic cells through exposure to a collection of growth factors known, iPSCs were initially developed in the early 2000s and have since been applied in a wide range of functions in both disease modelling and therapy development [44,45]. Originally the set of growth factors utilized for this reprogramming were known as Yamanaka factors, named after the researcher that initially demonstrated their potential; however due to the oncogenic nature of c-myc and klf4, additional factors were identified that could potentially be used in reprogramming human and mouse fibroblasts [46]. Hence, in addition to the original Yamanaka factors, OSKM, an additional cocktail of OCT4, SOX2, NANOG, and LIN28A (OSNL) was reported to reprogram mouse and human fibroblasts to iPSCs. LIN28 (a homologue of the Caenorhabditis elegans lin-28 gene) is a highly conserved RNA-binding protein and a master regulator controlling the pluripotency of embryonic stem cells. In concert with OCT4, and SOX2, the combination of NANOG and LIN28 dramatically enhanced the reprogramming efficiency and shortened the latency by greater than a week compared to the use of OSKM factors [47]. Even after the identification of the OSKM and OSNL cocktails over a decade ago, the reprogramming process remains to be inefficient and a lengthy process. As a result, the use of iPSCs is still not a common practice and studies are underway to evaluate the specific mechanism of transcriptional and post transcriptional control of human and mouse cell reprogramming processes [48]. The most common derivative tissues in the establishment of iPSC cultures are fibroblasts, cord blood cells, and peripheral blood mononuclear cells, which are then treated with a combination of Oct-3/4, Sox-2, Klf-4, and c-Myc to simulate conditions in ESCs and induce pluripotency [49,50]. In fact, the resulting pluripotent cell populations are highly similar to ESCs with respect to gene expression profiles and differentiation potential, leading to debates as to the equivalency of the two pluripotent cell types [51]. The induced pluripotency of these cells enables the new population to be capable of differentiation into all three germ layers, making this a uniquely potent tool in the field of tissue engineering and regenerative medicine. There are some concerns however, due to epigenetic changes and teratoma formation [52].

**Figure 2:**
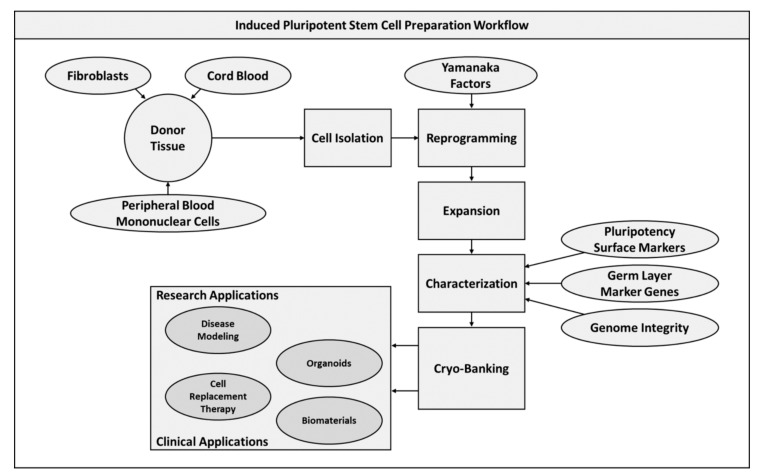
Induced pluripotent stem cell preparation workflow diagram.

In generating iPSCs cultures for future applications, it is critical to accurately characterize the transcriptomic and maturation potential of the expanded cells [53]. This ensures that the developed populations will function consistently given a particular set of parameters, whether this is seeding to a biomaterial or predifferentiating to a specific lineage utilizing a modified media. Additionally, an assessment of genomic integrity is essential to ensure that the reprogramming process has not deteriorated the capacity of cells to respond to their environment or stimulated oncogenic-like activity in populations. The later of these represents one of the major limitations of iPSC technologies, with propensity for teratoma formation preventing the use of human iPSCs (hiPSCs) in clinical human trials [54]. After establishing and expanding cultures, populations can then be cryo-preserved (Figure 2). This is possible at large scale given the ability to repeatedly reprogram cells, demonstrating the promise of this technology for developing clinically and commercially translatable therapies apart from oncogenic concerns.

Conversely, the use of iPSCs for other applications including disease modeling and chimeric research has shown high degrees of potential, as such functions are not impaired by these teratoma formation risks [7]. The ability to accurately model disease systems in vitro offers enormous promise for furthering development of effective therapeutics at expedited rates compared with current practices [55]. hiPSCs have been largely explored for this purpose with disease and drug screening models ranging across a variety of tissue types, as well as cancer development assessment [56,57,58]. Many of these model systems rely heavily on hiPSCs to generate organoid formations, which are cultured tissue masses that resemble particular organ systems in a simplified format. These organoids can be formed through different processes, depending on the target tissue, to generate uniformly distributed populations or randomly occurring tissue patterns, with many approaches utilizing coculture techniques to supply a vascularized network throughout constructs. Such modeling systems when coupled with the potential to implement predifferentiated iPSCs in targeted therapeutics demonstrates the transformative potential of these cells in regenerative medicine [59].

### 2.3. Embryonic Stem Cells

First derived from peri-implantation and pre-implantation embryos in 1998, human ESCs are pluripotent cells derived from the inner cell mass of blastocysts and capable of differentiating into any cell lineage of the three primary germ layers [60,61,62,63]. In 2007, human ESCs were still a relatively novel technology and were obtained by harvesting them primarily from ex utero embryos [64]. However, as the use of ESCs became increasingly prevalent, restrictions on how these cells could be obtained were implemented to address increasing ethical concerns. In the pre-implantation phase, the embryo undergoes changes in protein synthesis and metabolic fluctuations, developing from the fertilized zygote to a blastocyst [65]. Peri-implantation occurs until the blastocyst free-floating within the uterus initiates trophoblast differentiation, and is consequently most susceptible to disruption, following fertilization [66]. The blastocyst is the main cellular body which contains cells that form the outer trophectoderm, or trophoblast layer and the inner cells mass [66]. ESCs are primarily derived from the inner cell mass of pre-implantation embryos, with extensive research conducted exploring the differences in derivation and gene regulation [1]. Sagi et al. conducted research aimed towards the derivation of human ESCs, further highlighting the wide range of potential for application of these cells [67].

**Figure 3:**
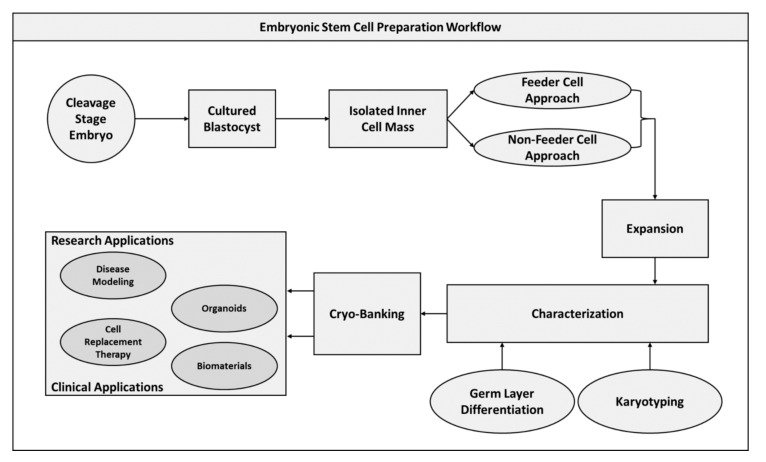
Embryonic stem cell preparation workflow diagram.

Isolated ESCs are traditionally cultured using feeder layers of fibroblastic cells which permit proliferation of these pluripotent cells [68]. More recently though, methods of ESC expansion that do not require feeder layers have gained attention as these techniques do not expose primary cultures to contaminating interaction with fibroblastic layers [69]. As culturing these undifferentiated populations in vitro can lead to an unstable genome and abnormal karyotype development during expansion, karyotyping is an essential characterization step for isolated cultures to verify genome integrity [60,70,71]. In addition to karyotyping, the capacity of expanded cells to differentiate to tissues of all three germ layers must be established, which is similar to assessment performed for the previously described iPSCs [72,73]. Following characterization, the expanded ESC populations can be cryo-preserved for future application (Figure 3).

As with iPSCs, ESCs have been utilized for a wide array of functions ranging from disease modeling to development of novel therapeutics. The generation of organoid formation through coculturing techniques provides an effective means of generating tissue that closely resembles native organ tissues and can therefore be used to evaluate disease progression or screen new drug formulae [74]. Additionally, tissue patches derived from these organoid 3D cultures have shown promising in vivo results when applied to a variety of rodent models for traumatic injuries and degenerative disease pathologies [75]. As compared with iPSCs, ESCs represent a steeply reduced risk of teratoma formation when applied in vivo, encouraging further exploration into utilization of these cells. Furthermore, the combinatorial use of ESCs seeded to biomaterials permits the ability to maintain implanted cells at the target site and stimulate specialized differentiation of populations to mimic native tissue function [76].

## 3. Applications of Stem Cells in Regenerative Medicine

The field of regenerative medicine can best be defined as a branch of medicine focused on repairing and restoring function of damaged tissue. Stem cells offer a powerful biological tool for developing such therapeutics as they can be readily extracted and expanded to bank large quantities of product while maintaining their versatility in differentiation potential. As such, stem cells have been utilized as a tissue engineering treatment mechanism for targeting injuries from trauma or disease for many different tissues. Tissue engineering arose in the 1950s after organ transplants were found to be inefficient due to shortages and alternative strategies were required [77]. Associates from Massachusetts General Hospital were first to spearhead the development of an alternative process to enhance the outcome of organ transplants, due to the issue of organ shortages for liver transplants [77]. This group of medical professionals theorized that combining elements of engineering and biological science could create a novel solution for increased organ transplant efficiency [77]. Thus, in May of 1987 the first liver, pancreatic, and intestinal tissues were engineered and presented to the American Pediatric Surgical Association [77]. Coupled with the discovery of stem cell science, the branch of regenerative medicine had planted roots within the medical community. While at first the idea received considerable backlash, within years the concept had spread worldwide [77].

The application of stem cells in conjunction with scaffold technologies has been observed to both maintain cells at the site of implantation and to offer modulatory functions for cell fate [78]. Ideal biomaterials for such usage must demonstrate several key attributes that govern the interaction of cells with the platform. Perhaps the most critical of these characteristics are a material’s cytocompatibility and biocompatibility, indicating the toxicity associated with that material upon exposure to cells in vitro and in vivo. These aspects can be assessed in vitro through the use of cell viability stains and general cell morphological observation after exposure to a material; however, in vivo assessment may be more challenging, as compatibility issues may present as a severe inflammatory response or tissue necrosis. Materials that illicit such responses or are observed to be toxic to cells in culture thereby cannot be considered candidates for therapeutic applications despite any other attributes. Apart from this critical attribute, the biodegradability of a material may also determine its suitability for a given application. A nondegradable material will inherently be a permanent implant without additional surgery. Therefore, an ideal implanted structure can be degraded at a rate similar to the ingrowth of surrounding tissue, which would result in the elimination of the scaffold coinciding with the complete regeneration of tissue at the site. This degradability will be largely dependent on the constitutive materials of the scaffold and will vary depending on intended target tissue and the level of structural support required at the site. To this end, an effective biomaterial should closely match the mechanical properties of existing tissue to avoid compromising the native architecture. These mechanical properties include strength, toughness, elasticity, structural support (stability), and stiffness. Furthermore, an ideal biomaterial should demonstrate some basal degree of antibacterial properties to aid in preventing site infection that could lead to graft rejection [79]. Surgical operations pose an inherent risk of exposing patient tissues to bacterial infections, which can result in further health complications and death, to minimize these risks biomaterials that have innate bactericidal properties or that work well with common antibiotics offer attractive options. Lastly, a vital aspect of tissue repair and regeneration is the ability to stimulate the intended tissue formation at the site of interest. Therefore, it is important that a biomaterial is capable of either inducing or facilitating the necessary gene expression for on-board cells to correctly develop into appropriate tissue.

A material implanted with MSCs includes additional requirements for successful tissue regeneration. The cell–material interface becomes a platform for bioactivity, eliciting the cellular responses of adherence, proliferation, differentiation, and ECM synthesis. Cellular adherence to the material is essential to establishing an effective material platform that can then be utilized as a therapy. Seeded stem cells should be able to attach and proliferate along the provided substrate to ensure that the loaded construct will maintain the biological payload post-implantation. Only once adhered can cell-to-cell communication then facilitate both proliferative and maturation functions. This initial attachment is regulated by key integrin proteins, which are transmembrane receptors that form focal adhesion clusters. These focal adhesions connect the cell’s actin cytoskeleton with the ECM. Within focal adhesions is focal adhesion kinase, which signals adherence of cells to each other and their environment (i.e., a biomaterial, tissue culture plate, etc.). Additionally, the surface topography of a material can substantially impact the interaction with cells. Varying degrees of surface roughness provide differing architectural landscapes to which cells can anchor and are correlated with not only the adherence and proliferation of undifferentiated cells, but also the subsequent proliferation maturation characteristics [80]. Other surface features that may affect the cell–material interaction include surface charge, surface chemistry, wettability, and surface density of cell-binding ligands [81].

This section explores the extensive utilization of stem cells in the field of tissue engineering to develop effective therapies for injuries and diseases. Specifically, the research and clinical application of stem cells, both as a stand-alone treatment and in conjunction with biomaterials, will be examined with respect to the target tissue, including mesodermal, ectodermal, and endodermal lineages, for regenerative intervention.

### 3.1. Mesodermal Applications

#### 3.1.1. Bone

Bone represents a unique challenge to the design of therapeutics for damaged tissue due largely to the highly dynamic nature and the mechanical force demands of the native architecture. For this reason, the development of cell- and biomaterial-based treatments for bone are varied in both approach and effectiveness. Regarding ideal biomaterials for such applications, the functional characteristics osteoinduction, osteoconduction, and osteointegration are the primary attributes associated with suitable osteogenic constructs. Osteoinductive and osteoconductive functions are related to the capacity of a material to stimulate undifferentiated cells toward an osteogenic lineage and the potential for cellular proliferation on structure surfaces respectfully [15,82]. Osteointegration refers to the ability of an implant to interact and integrate with existing tissues. Furthermore, the mechanical attributes required for an effective biomaterial must resemble those observed in the native tissue, which will vary based on the injury site with major differences emerging between load and nonload bearing structures. The implementation of these bone scaffolds can be limited in impact due to the need to recruit local cell population, which can be minimal in certain disease models. Therefore, the application of stem cell populations in conjunction with constructs may offer an enhanced regenerative capacity for treating these complex injuries. 

Cell-based therapies utilizing MSCs, iPSCs, and ESCs have been largely implemented in various strategies for bone tissue engineering. MSCs derived from adipose, bone-marrow, iPSC, and ESC origins have demonstrated the potential to differentiate to osteo-progenitors capable of osteoblastic functions when stimulated either by growth factors or osteogenic platforms [83,84,85]. Furthermore, the incorporation of coculturing techniques with endothelial-like cells in addition to incorporation of calcium phosphate-based materials have offered the ability to generate vascularized implants, which can substantially improve the osteointegrative potential of the treatment [86,87]. Similar strategies can be utilized with other osteogenic platforms, such as bioactive ceramics, biodegradable polymers, biodegradable metals, and biological substrates, to allow for modulation of mechanical properties through micro- and ultra-structural design [88]. For nonload bearing injury sites, the application of MSCs seeded to elastic and porous material implants has shown that undifferentiated cells alone are capable of stimulating tissue repair [89]. This is particularly of interest for cranio-maxillofacial injuries as scaffolds can be tailored for cell- and/or drug-loading functions without concerns of the material being compromised due to mechanical forces. For such therapies, highly bioactive material designs like those derived from the extracellular matrix (ECM) have proven to be effective regenerative technologies, due to the rich assortment of vital proteins available in these materials for seeded undifferentiated cells. The application of MSCs in combination with these bioactive substrates have demonstrated enhanced reparative capacity, such as the implementation of MSC cell sheets [90]. 

In addition to MSCs, iPSCs have been largely explored in bone tissue engineering, with the primary advantage being the ability to expand these cells at a greater scale compared with MSCs. The expanded cells can readily be driven toward MSC, and specifically osteogenic, maturation and then implemented in a wide variety of biomaterials, thereby offering a personalized therapeutic option [91]. In a study conducted by Wang et al., such iPSC cultures were utilized in conjunction with a calcium phosphate ceramic to generate an injectable osteogenic platform with a potent bioactive component [92]. Similarly, another study applied iPSCs in a self-assembling biomaterial for repairing calvarial defects in rodents [93]. As previously mentioned, particular attention has been given to developing osteogenic platforms that can stimulate angiogenic activity or have been prevascularized through coculturing methods. Several studies have approached this concept, utilizing both MSCs and iPSCs in combination with endothelial-like cell populations [94,95].

#### 3.1.2. Cartilage

The avascular nature of cartilage poses a challenge in development of effective therapeutics for tissue damage as implants may be unable to integrate with the native environment. Additionally, inflammatory reactions to implanted materials and injected therapies may result in further damage to tissue surrounding the original injury. Diseases associated with cartilage can lead to the breakdown of these force absorbing tissue pads, resulting in bone-to-bone contact and high degrees of pain for afflicted individuals. Osteoarthritis represents one of the most common of these diseases, which is characterized by chondrocyte degeneration due to reduced expression of growth factors, mitochondrial deterioration, inflammation, oxidative stress, and extensive ‘wear-and-tear’ of protective cartilage surrounding bones [78]. Articular cartilage naturally displays a limited capacity for self-renewal due to its lack of blood vessels and hypocellularity, with articular lesions potentially resulting in irreparable damage particularly in defect sizes that exceed 3mm [96]. Current means of treatment are the utilization of modifying osteoarthritis drugs (DMOADs) or extensive surgeries such as subchondral drilling, microfracturing, and abrasion arthroplasty. Such methods offer only temporary relief in many cases, while exposing patients to heightened risks associated with surgical operations. For younger patients with osteoarthritis, finding prostheses that fulfill the physiological demands of their more energetic lifestyles is challenging. Therefore, regenerating tissue, with stem cells, could be the future for remedying osteoarthritis in patients of all ages and abilities [78]. Additionally, stem cell-based therapies may offer an enhanced therapeutic alternative for damage caused by intervertebral disc degeneration (IVD), which is characterized by the decomposition of fibrous cartilage between the spinal bones resulting in severe back pain and limited mobility. As the common surgical methods for treating IVD constitute a heavy financial burden and a personal health risk, alternative therapies have garnered increased attention [97]. Cellular therapies have the potential to stimulate matrix synthesis and modulate cytokine recruitment to prevent damaging inflammation, thereby providing a treatment option with reduced pain levels and increased motility.

The application of MSCs via injection has been demonstrated as a potent means for treating damage to articular cartilage caused by trauma or chronic injury. However, the use of bone-marrow-derived MSCs has been shown to induce fibrocartilaginous repair, which is not ideal for restoring cartilage tissue functionalization [96,98]. A wide assortment of biomaterials including hydrogels and bioactive collagen matrixes have been implemented in combination with undifferentiated cells to provide a substrate for implanted cells. Several studies have examined the clinical applications of these cell-material therapeutics for the treatment of osteoarthritis, with on-going clinical trials still in early phases. Such methods have been observed to demonstrate promising results in cartilage regeneration in these preclinical and clinical settings, in particular the use of natural hydrogels including chitosan and alginates [99].

In addition to the application of MSCs, iPSCs and ESCs have seen utilization in many studies examining their capacity for stimulating cartilage repair in articular lesions and microfractures [100]. iPSCs and ESCs are capable of readily differentiating to a chondrocyte lineage and secreting key proteins for stimulating repair. Secretion of exosomes by these cells may further offer a protective ability against inflammatory agents at the implantation site [101]. The major limitation of applying iPSCs to such therapies is a lack of uniformity in the differentiation of cell populations and inherent oncogenic risks related to these cell types. Therefore, methods to address these concerns are essential for the future of iPSC-based cartilage treatments, which may fall to optimized coculture techniques [102]. An alternative technique is to utilize iPSCs to derive MSC cultures that can then be further differentiated to chondrocytes. As observed in a clinical trial study in South Korea, pluripotent-derived MSCs demonstrated a safe and effective means for regenerating articular cartilage [103,104]. ESCs have also been explored with similar methodologies, where pluripotent cells have been expanded and subsequently differentiated to MSCs and chondrocytes. Similar to iPSCs however, transplanted ESCs have demonstrated a potential to form teratomas and therefore may be more suited for applications in which cells are predifferentiated to a specific tissue type prior to transplantation [105,106].

#### 3.1.3. Muscle

Muscle tissue is comprised of multinucleated contractile cell bodies connected to a specialized ECM known as the basal lamina and is responsible for facilitating many crucial functions including structural motion via skeletal muscle and blood pulsatile pumping via cardiac muscle [107]. These finely tuned actions can be steeply compromised through damage caused by either traumatic injury or disease, with cases such as cardiac diseases posing severe risks to life of patients. The development of effective therapeutics for muscle tissue is therefore a focus of regenerative medicine though both cell-based and biomaterial-based mechanisms, and stem cells have recently become a promising candidate for potential treatments [78]. The predisposition of MSCs to differentiate into the cell type from their originating tissue has spurred interest into utilizing multipotent muscle-derived stem cells (M-MSCs) for such therapies. Despite a standardized isolation technique for M-MSCs not yet being developed, there are two major methodologies currently in place. The first of these techniques is known as preplating in which digested muscle tissue is seeded to tissue culture plates with periodic media changes to isolate cells that adhere more slowly, which ideally should be primarily M-MSCs. The second method utilizes cell sorting techniques to isolate uniform populations based on fluorescent surface markers [108]. A recent study by Čamernik et al. demonstrated that this isolation of M-MSCs can be effectively performed from osteoarthritic patients through minimal tissue, with only approximately 5–10 g required [109].

Other MSC sources have also been explored, including BM-MSCs, gingival-derived MSCs, and tonsil-derived MSCs, and have demonstrated myogenic potential; however, these cells often require cell-to-cell interactions via coculturing techniques with primary myoblasts and implementation of scaffold technologies [110,111,112]. A study conducted by Chiu et al. observed an increased regeneration of myofibers after application of BM-MSCs, with and without a polymeric gelling solution, to a rodent muscle contusion injury [113]. In addition to treatments for skeletal muscle, MSCs have demonstrated effectiveness in applications for cardiac tissue damage, with one study showing a regulatory relationship between MSCs and macrophages following a myocardial infarction [114]. Preclinical studies utilizing MSCs for cardiac-centric therapies have attempted combinatorial applications with ESCs and biomaterials, to improve biofunctional capacity of the injection and MSC retention, respectively [115]. Such methods have shown promise in stimulating enhanced reparative function of damaged tissue as well as reduced scar tissue formation [116].

The implementation of biomaterials from natural and synthetic origins have been applied in combination not only with MSCs, but also with iPSCs and ESCs in attempts to develop specialized culture tissue patches for effective therapeutics in both skeletal and cardiac muscle. One such approach makes use of decellularized ECM scaffolds, which maintain a rich-protein collection capable of facilitating cell–material interactions, in combination with ESC-derived MSCs for treatment of volumetric muscle loss [111,117]. Other approaches employ iPSCs as a means of generative large-scale populations of well-characterized cryopreserved cell banks that can be applied in a variety of functions and foster the development of both clinically and commercially translatable therapeutics [118,119,120]. iPSCs have further demonstrated a wide array of potential, such as the synthesis of vascular rungs from iPSC derived smooth muscle and the development of a novel therapy for Duchenne muscular dystrophy [121,122]. Stem cell applications associated with mesodermal tissue are listed in Table 1.

### 3.2. Ectodermal Applications

#### 3.2.1. Nerve

The repair and restoration of function for nerve tissue has presented one of the most daunting challenges for the field of regenerative medicine. This is largely due to the nonproliferative nature of mature neuronal cells and the complex nature of the tissue structure. The substantive impact on quality of life that traumatic and disease-related injuries to these systems further highlights the imperative demand for effective reparative therapies [123]. The utilization of undifferentiated cells with and without biomaterials have presented promising avenues for such treatment development, with a recent focus on methods for addressing prevalent neurological diseases including Alzheimer’s and Parkinson’s. Both of these diseases represent degenerative pathologies in which the afflicted individual will gradually lose nerve system-related functions and eventually lead to death. Stem cell therapies commonly implemented for Alzheimer’s disease have centered on the application of MSC-, iPSC-, and ESC-derived neural stem cells (NSCs) in order to provide the injury site with a predifferentiated cell population. Clinically, predifferentiation of ESCs into NSCs and medial ganglionic eminence-like progenitor cells, which are capable of maturation into stimulating and inhibitory neuron types, have led to increased function in spatial memory and learning abilities [124].

The flexibility of stem cell-based therapies for neuronal degenerative diseases demonstrate an attractive prospect with increased attention being given to the development of iPSC and ESC-derived dopaminergic (DA) progenitor cells that can be utilized in therapeutics for Parkinson’s disease. These DA cell populations show enhanced survivability after implantation at the target site and have seen application in several preclinical and clinical trials for the disease [125,126,127]. The potential of these cultures can be further enhanced by combinatorial use with biomaterials, such as in a study by Tasnim et al. that utilized graphene foams to stimulate exposed MSCs toward DA neurons [128]. Focusing on clinical translatability, some studies implement iPSCs for their ability to be expanded at large scale. These banked undifferentiated cells can then be differentiated to DA neurons for standalone application or be complimented by biomaterials that will stimulate differentiation and growth in a defined manner [129,130]. The use of ESCs in a similar method has been implemented, with the objective to remove the inherent oncogenic risks associated with iPSCs [131].

#### 3.2.2. Skin

The repair and restoration of skin has been a long-time focus of tissue engineering, with many cell- and biomaterial-based therapies emerging. Dermal injuries are a common complication ranging from lacerations, disease-related degeneration, and burn-associated injuries. Furthermore, many cases not only represent severe localized damage, but also may cover large surface areas. These cases, such as severe burns or extensive tissue damage, often require large grafting technologies to effectively address the injured tissue. The gold standard for such procedures is the use of autologous tissue taken from other surfaces of the body; however, with burn victims this may not be a viable option. Biomaterials alone have offered some degree of effect but leave much to be improved. The addition of stem cells as a means for stimulating repair therefore demonstrates a promising alternative treatment method [132,133].

Implementation of MSCs has been extensively explored for generating cellular sheets that can be applied as grafting technologies [134]. These technologies are further enhanced through coculturing techniques with endothelial-like cells to develop prevascularized cell construct sheets that display improved integration with the native tissue and survivability of the graft [135]. Similar coculture methods have been utilized with iPSC-derived epidermal patches to synthesize highly vascularized scaffolds for full-thickness skin injuries [136,137]. Studies taking this approach generally utilized well-characterized and expanded iPSC populations to derive MSC with angiogenic and keratinogenic properties that can enhance grafting effectiveness [138]. A recent study by Liu et al. employed iPSCs as a means to generate cultures of induced-melanocytes to develop potential therapies for vitiligo [139]. Methods similar to those used for iPSCs are also implemented for ESCs to develop MSC, fibroblasts, and keratinocytes [140,141,142]. Stem cell applications associated with ectodermal tissue applications are listed in Table 2.

### 3.3. Endodermal Applications

#### 3.3.1. Liver

The liver is the largest internal organ, playing a vital role in detoxification, metabolic, and immunologic processes. If damaged, the liver is able to regenerate itself using native cells, but this is clinically difficult to predict, often requiring medical technology to stimulate regeneration and restore liver function. Liver disease is the 12th leading cause of death in the U.S., and therefore a focused effort has been made to manipulate the self-regenerative properties of the liver, thereby decreasing the need for liver transplants [143]. These include the injection of mature hepatocytes, implantation of predesigned cellular constructs, and the use of engineering tissues as in vitro models. A major barrier in the progression of cellular-based therapies for liver tissue is the complexity of the organ. Hepatocytes, the functional cells of the liver, are organized in parallel cords surrounded by extracellular matrix within the liver’s multicellular structure. Between these cords are additional cell types, such as Kupffer cells and biliary ductal cells, that facilitate interaction with many growth factors, hormones, and nutrients that are transported and deposited through the hepatic artery and portal vein located in the upper right-hand quadrant of the abdomen [143].

Cellular-based treatments for liver disease also are limited, despite their enhanced regenerative potential, due to the restricted capacity for proliferation and functional maturation of hepatocyte-like cells [143]. Effective expansion of undifferentiated cell culture to develop therapeutic doses, and subsequent differentiation of these cultures to mature and functional hepatic tissue constitutes an attractive alternative to current transplant options. In an examination of a lethal hepatic failure model in nondiabetic mice both MSCs and functional hepatocytes derived from MSCs were utilized to assess their capacity to restore the damaged tissue. It was observed that, while both cell population were capable of rescuing the injured tissue, intravenous application of MSCs without prior differentiation demonstrated preferential results [144]. This was credited to an enhanced resistance of the undifferentiated cells to reactive oxidative species, thereby correlating to a greater survivability at engraftment sites of the damaged liver [144]. However, a later study further examining the application of MSCs in treating liver fibrosis demonstrated that predifferentiation of MSCs to functional hepatocytes may be a viable and effective therapeutic method without the risk of cells differentiating to cell lineages that might aggravate the disease condition. Cells applied through intravenous, intraperitoneal, and intrahepatic injection were evaluated though both gene expression and histological techniques to determine their impact on tissue. The findings from this analysis yielded similar results to the previously described study with the implanted cells providing protective effect through engraftment and stimulation of essential growth factors at the site of injury [145].

iPSCs have also proven to be of great interest in the development of effective alternative methods for treating hepatic diseases. One of the key advantages of applying iPSCs as compared with the previously described MSCs is the potential form complex 3D organoid structures. An example being the culturing of functional and vascularized organoids conducted in a study by Takebe et al. in which iPSC-derived liver buds (iPSC-LBs) were formed in vitro through the coculturing of iPSC-derived hepatic cells, human umbilical vein endothelial cells (hUVECs), and MSCs [146]. These immature tissue constructs can then be implanted in vivo with the objective of integration between intra-construct and native vasculature, thereby promoting the survival of the matrix. Further refinement of this technology with the primary goal being the development of its clinical translatability resulted in the formation of iPSC-LBs utilizing only iPSC-derived cell cultures. By building of the ever-developing field of organoid research, the described study employs methods of characterization and scalable-manufacturing that offer the potential for an effective transplantable tissue patch [147]. In a similar approach, another study utilized iPSCs to form hepatocyte sheets for treatment of acute hepatic damage. The cultured tissue sheets were observed to successfully rescue liver damage caused by infusion of carbon tetrachloride, which is a common modality for simulating acute liver failure in mice [148]. Though not as commonly utilized in the development of clinically translatable treatments for hepatic damage, ESCs have seen some implementation in a similar form as the described iPSC-derived organoid transplants. A study by Wang et al. demonstrated the potential to generate expandable hepatic organoids from ESCs based on exposure to tailored media additives [149]. The objective of this study was to form consistent organoid structures while avoiding the oncogenic risks commonly associated with iPSCs and that would be effective in treating hepatic alcohol-associated damage.

#### 3.3.2. Vasculature

Formation and repair of intricate channels within the vascular system depend on the angiogenic potential of local undifferentiated cell populations. The differentiation of these progenitor cells to mature endothelial cells provide for the lining of vessels, the structure of which must demonstrate both elasticity and resistance to shear forces. Injury to these structures due to obstruction or trauma can result in massive downstream tissue damage from ischemic and hypoxic conditions. Additionally, vascular grafting can be essential in operations such as cardiac bypass treatments, in which the patient’s blood supply to and from the heart are occluded, and as of currently the gold standard for these treatments is the use of autografts from the patient. The application of stem cells and biomaterials in conjunction with stem cells have therefore become an attractive option for future therapy options.

MSC, iPSC, and ESC have been implemented in designing therapeutic strategies for vascular tissue engineering technologies, with a key aspect being the stimulation of expression for key angiogenic-associated markers including CD31, CD34, and vascular endothelial growth factor (VEGF). These induced progenitor cell populations can then be utilized in synthesis of tissue patches closely resembling endothelial lining surfaces that can then be applied as graft technologies [150]. Combining such cells with biomaterials designed to act as substrates for vascular grafts can further enhance the potential regenerative capacity of these technologies [151]. Apart from the direct application of such materials and techniques for regenerative vessels for grafting, the uniquely vital nature of vasculature for most tissue types in the form of nutrient and oxygen delivery means that there is a need for implementing angiogenic features in many other tissue engineering applications [152]. For example, the success of scaffold technologies such as those for bone or dermal tissue hinge greatly on the potential of the biomaterial to stimulate vascular development or infiltration, as this will supply the newly forming tissue with vital nutrients.

A common approach for stimulating angiogenic functions both in in vitro cultures and in scaffold technologies is the coculturing of undifferentiated cells from various sources with endothelial-like cells [153]. Commercially available human umbilical vein endothelial cells (hUVECs) and other endothelial progenitor cells can readily be cocultured with MSCs, iPSCs, and ESCs to promote the formation of angiogenic fractions within the culture population. Combining such cocultures with biomaterial platforms then permits the ability to stimulate early vascularization through a implanted matrix, enhancing the survivability and thereby functionality of that scaffold. While this concept has been applied in regenerative applications for several tissue types, some of the most noted examples have been observed in bone tissue engineering and nerve regenerative therapies [86,154]. A study examining the coculture of osteogenic differentiating MSCs and endothelial cells seeded to a collagen hydrogel within a dynamic bioreactor environment demonstrated increased expression of key proteins associated with neovascularization and enhanced osteogenic functions [155]. A variety of osteoblastic cells have been utilized in combination with endothelial-like cells to further promote similar pro-angiogenic and pro-osteogenic capacities in both 2D and 3D systems [156]. Continued efforts to further optimize coculture conditions and implementation in scaffold technologies therefore poses a substantial benefit to the development of effective biomaterials with improved integrative abilities.

#### 3.3.3. Gastro-Intestinal

The gastro-intestinal (GI) system represents a complex collection of highly functional hollow structures responsible for nutrient processing, transportation, and excretion. Damage to these various tissue linings can have severe impacts on health depending on the location of injury. In particular, the intestinal tract has been the focus of regenerative medicine efforts due to the prevalence of intestinal diseases in the population, with diseases such as irritable bowel disease (IBD) resulting from inflammation in the mucosal lining. Such diseases are further complicated by the presence of micro-organisms of the natural gut biota, which can lead to infection or inflammatory responses in the surrounding environment of the injury. As the tissue lining for organs of the GI system have innate elasticity and protective luminal surfaces, development of stem cell and biomaterial therapeutics has been slow in progress with a wide variety of materials being implemented both with and without bioactive additives [157]. Among these studies, the application of adipose-derived MSCs encapsulated within silk fibrin microspheres demonstrated enhanced repair of a rodent urinary tract defect model, with both increased burst pressure and decreased lumen diameter [157,158]. The combination of similar cell-based and organoid methods with polymeric sheath technologies have also been attempted with promising results [159]. Additionally, 3D printing, organoid, and decellularization techniques have demonstrated the possibility of constructing full-organ transplantable biomaterials [160,161]. 

Application of MSCs in developing treatments for various GI-related diseases have been widely explored, with over 200 registered clinical trials in progress or completed, due to the simplicity of extraction and expansion for these cells as well as promising therapeutic effects. A review examining the impact of MSCs on chronic inflammatory fistulizing and fibrotic diseases demonstrated that these cells are capable of promoting accelerated repair of tissue compromised by radiotherapy [162]. The described studies in this review utilized an intravenous approach for cell application and noted that inflamed tissue may stimulate enhanced honing of MSCs to target sites [162]. Alternative delivery mechanisms have been assessed for MSCs, with some of the most promising being either biomimetic synthetics or biologically derived substrates. An example being the use of small intestine submucosa (SIS) as a biocompatible matrix for improved enrichment of target tissue with implanted MSCs, with the composite gel demonstrating heightened survivability compared to conventional intravenously delivered cells [158].

More recent research attention has been given to implementation of iPSC-derived MSCs for repair of intestinal tissue due to the potential to expand large quantities of these cells without sacrificing the effectiveness observed in MSCs from past studies. Utilizing this method for developing MSC population ideally avoids the general concerns related with primary MSC lines regarding loss of stemness over multiple cell culture passages, since iPSCs operate via reprograming of somatic cells. Histological evaluation of treated tissue from a well-defined colonic mouse model demonstrated that the iPSC-derived MSCs were capable of effectively restoring the functionality of the damaged tissue [163]. Similarly, promising results have been shown with the recellularization of decellularized tissue architectures with predifferentiated iPSCs. This cell-seeded graft technology, when applied in a rodent in vivo model, was found to function similar to mature intestinal tissue [164]. Furthermore, organoid research has been a recent focus for the development of regenerative therapeutics for intestinal tissue injuries. Though much of this research has utilized iPSCs, implementation of ESCs has also seen promise, while reducing the oncogenic risks associated with iPSC maturation. Such work can be observed in a study assessing the potential of both biological and polymeric scaffold patch designs seeded with ESC-derived organoids for repair of intestinal tissue damage. It was demonstrated that the examined polymeric scaffolds were highly effective in promoting cellular attachment and proliferation throughout the matrix and stimulated the formation of tissue architecture resembling mature mucosal lining of the small intestine [165].

#### 3.3.4. Lungs

The respiratory system offers a unique challenge to regenerative medicine, as lung tissue is regularly subjected to potential impurities during gas exchange mechanisms. These can range from carcinogenic agents, as might result from smoking, to pathogens that are able to establish foothold, and resulting inflammation or infection can lead to chronic disease formation or death. Currently, treatment methods for lung tissue following such instances are limited in effectiveness as they are not capable of repairing the resulting scar tissue associated with many of these diseases, including pulmonary fibrosis and chronic obstructive pulmonary disease (COPD). Stem cells offer a promising therapeutic mechanism for such injuries as they provide a means for not only repairing the tissue but restoring full functionality to the system. This development of effective treatments is all the more pressing in the wake of the outbreak of severe acute respiratory syndrome coronavirus 2 (SARS-CoV-2 or COVID-19), which has impacted the lives of over 40 million individuals worldwide and resulted in the death of over 1 million people in the year 2020 alone (https://coronavirus.jhu.edu/map.html) [166].

The demand for such regenerative therapies has further stimulated research into the applications of undifferentiated cells in treating these complex diseases, with a wealth of preclinical studies examining the potential of unipotent endothelial progenitor cells (EPCs), MSCs, iPSCs, and ESCs [18]. MSCs have demonstrated particular potential in the treatment of inflammatory diseases in the lungs and are being explored in a phase-II clinical trial as a therapy for COPD patients [18,167]. One of the most critical obstacles to overcome for these MSCs-based treatments is the survivability of the cells and the localization of them to the target tissue. Multiple approaches have been utilized to extend the survival of delivered cells within the injured tissue, including preconditioning of the cells to enhance homing capacity as well as durability and the genetic manipulation of cells to induce desired characteristics [168], yet as of currently these method have only demonstrated limited improvements. Despite this, there are a growing number of preclinical studies examining the application of MSCs for treatments, particularly with regard to virus-based diseases. Among these are a number of cell-based trials focused on utilizing these undifferentiated cells as a therapy for COVID-19, though these are still in early trial phases [169]. Apart from MSCs, both iPSCs and ESCs are also considered promising candidates for future therapeutics, with research focusing primarily on predifferentiating these cells to mature to lung epithelium lining [170]. However, one of the major complications with this approach is that the resulting tissue after differentiation still does not represent mature epithelium and therefore unable to fulfill functional roles [171]. Furthermore, the protocols implemented to stimulate this maturation can be inconsistent among research groups leading to mixed and conflicting data with respect to resulting differentiated cells. Such challenges may be addressed through continued assessment of iPSCs and ESCs for this therapeutic application and though development of a more standardized differentiation protocol. Stem cell applications associated with endodermal tissue applications are listed in Table 3.

## 4. Conclusions

The implementation of stem cells for regenerative medicine applications is an ever-expanding focus of the field of tissue engineering, with many promising studies conducted across multiple tissue types and organ systems. MSC, iPSC, and ESC populations represent a uniquely powerful set of biological tools for addressing both traumatic and disease-related injuries given their capacity to be expanded, stored, and utilized for a variety of tissues. Extensive research for vital systems in all three germ layers has demonstrated that cell-based therapeutics utilizing these undifferentiated populations offers the potential for the development of novel and highly effective treatments. Despite this subfocus in the field of tissue engineering being more advanced currently in applications relating to bone and cartilage, the relevance of these techniques can readily be applied to all tissues, as demonstrated by the recent heightened exploration and development of therapeutics for the respiratory system. Furthermore, the combinatorial application of MSCs, iPSCs, and ESCs with natural and synthetic scaffold technologies can enhance the reparative capacity and survival of implanted cells at the target location. Continued efforts to generate more standardized approaches for these cells may provide improved study-to-study variations on implementation, thereby increasing the clinical translatability of cell-based therapeutics. The availability of these cell types to researchers through several commercial sources, as well as the established protocols present in literature, provides a conducive environment for technological advancements in the field of regenerative medicine that can drastically enhance the quality of care and life for individuals suffering from degenerative diseases or traumatic injury.

## Figures and Tables

**Table 1:** Summary of mesodermal layer stem cell applications.

Mesodermal Stem Cell Applications					
Target Tissue	Utilized Stem Cell Populations			Biomaterial(s) Utilized	Reference(s)
	MSCs	iPSCs	ESCs		
**Bone**	X	X	X	Yes	[83,86]
	X			Yes	[84,87,88,89,90]
	X			No	[85]
		X		Yes	[91,93]
	X	X		Yes	[92,95]
	X	X		No	[94]
**Cartilage**	X			No	[96,98]
	X	X		Yes	[99,103]
	X	X		No	[100]
	X	X	X	No	[101]
		X		No	[102]
	X			Yes	[104]
			X	Yes	[105]
**Muscle**	X	X		Yes	[107]
	X			No	[108,109,110,112,114]
	X			Yes	[111,113,115,116]
			X	Yes	[117]
		X		No	[118,119,121,122]
		X		Yes	[120]

**Table 2:** Summary of ectodermal layer stem cell applications.

Ectodermal Stem Cell Applications					
Target Tissue	Utilized Stem Cell Populations			Biomaterial(s) Utilized	Reference(s)
	MSCs	iPSCs	ESCs		
**Nerve**	X			Yes	[123,128]
	X	X	X	No	[124]
		X		No	[125,126]
		X	X	No	[127]
		X		Yes	[129]
			X	Yes	[130]
			X	No	[131]
**Skin**	X	X	X	Yes	[132]
	X			Yes	[133]
	X			No	[134,135]
		X		Yes	[136,137]
	X	X		No	[138]
		X		No	[139]
			X	No	[140]
	X		X	No	[141]
	X	X	X	No	[142]

**Table 3:** Summary of endodermal layer stem cell applications.

Endodermal Stem Cell Applications					
Target Tissue	Utilized Stem Cell Populations			Biomaterial(s) Utilized	Reference(s)
	MSCs	iPSCs	ESCs		
**Liver**	X			No	[144,145]
		X		No	[146,147,148,161]
			X	No	[149]
**Vasculature**	X	X	X	Yes	[86,150,152]
	X			No	[153,154]
	X			Yes	[155,156]
**Gastro-intestinal**	X			Yes	[158]
		X		No	[161]
	X			No	[162]
	X	X		No	[163]
		X		Yes	[164]
			X	Yes	[165]
**Lungs**	X	X	X	No	[18,170]
	X			No	[166,167,168]
			X	No	[169]
